# Evidence-based clinical practice guidelines for the management of perioperative hypothermia: Systematic review, critical appraisal, and quality assessment with the AGREE II instrument

**DOI:** 10.1016/j.amsu.2022.103887

**Published:** 2022-05-31

**Authors:** Mohamed Ali Babiker Mohamed, Wael Ahmed Abdelwahab Abdelkarim, Mohamed Abdulmonem Salih Aabdeen, Tarig Hassan Elobid Ahmed, Hassan H.H. Sarsour, Ahmed M. El-Malky, Yasser S. Amer, Nuha alsaleh, Rakan I. Nazer

**Affiliations:** a Leeds University Teaching Hospital, Plastic and Reconstructive Surgery, Leeds, United Kingdom; b Department of Obstetrics and Gynaecology, King Saud University Medical City, Riyadh, Saudi Arabia; c Paediatric Surgery Department, Paediatric Urology Division, Leeds University Teaching Hospital, Leeds, United Kingdom; d Trauma and Orthopaedic Department, Manchester Royal Infirmary Hospital, Manchester, United Kingdom; e Nutrition Officer at Mercy Corps Europe, Kadugli, Sudan; f Public Health and Community Medicine Department, Theodor Bilharz Research Institute, Academy of Scientific Research, Ministry of Higher Education, Cairo, Egypt; g Morbidity and Mortality Review Unit, King Saud University Medical City, Riyadh, Saudi Arabia; h Pediatrics Department, King Khalid University Hospital, Riyadh, Saudi Arabia; i Research Chair for Evidence-Based Health Care and Knowledge Translation, King Saud University, Riyadh, Saudi Arabia; j Clinical Practice Guidelines and Quality Research Unit, Quality Management Department, King Saud University Medical City, Riyadh, Saudi Arabia; k Alexandria Center for Evidence-Based Clinical Practice Guidelines, Alexandria University, Alexandria, Egypt; l Adaptation Working Group, Guidelines International Network, Perth, Scotland, United Kingdom; m Department of Surgery, King Saud University Medical City, King Saud University, Saudi Arabia; n Cardiac Science Department, King Fahad Cardiac Science Center, College of Medicine, King Saud University, King Saud University Medical City, Riyadh, Saudi Arabia

**Keywords:** Practice guidelines, Critical appraisal, AGREE II instrument, Perioperative hypothermia, Quality assessment

## Abstract

Inadvertent perioperative hypothermia is considered an emergency life-threatening situation. Clinical practice guidelines (CPGs) on how to manage hypothermia, based on evidence and expert opinions, could save lives. This systematic review assessed and compared the most recently approved international CPGs with the AGREE II instrument. We searched international bibliographic databases to identify relevant guidelines for managing perioperative hypothermia. Four independent reviewers (consultant anesthesiologists) critically appraised the selected guidelines with the AGREE II instrument. We analyzed inter-rater agreement and calculated an intra-class correlation coefficient (Kappa). We identified five CPGs for perioperative hypothermia that were eligible for critical appraisal. These CPGs were issued by the National Institute for Health and Care Excellence (NICE-2016); the American Society of Peri-Anesthesia Nurses/Agency for Health Care Research and Quality (ASPAN/AHRQ-2006); the University of Southern Mississippi (USM/CPG-2017); The University Assistance Complex of Salamanca (UACS/CPG-2018); and the Justus-Liebig University of Giessen (UKGM/CPG-2015). The overall assessments of NICE-2016 and ASPAN/AHRQ-2006 scored >80%. These results were consistent with high scores achieved in the six domains of AGREE II: (1) scope and purpose, (2) stakeholder involvement, (3) rigor of development, (4) clarity of presentation, (5) applicability, and (6) editorial independence domains. The NICE-2016, ASPAN/AHRQ-2006, and USM/CPG-2017) scored, respectively, 94%, 81%, and 70% for domain 3, 91%, 87%, and 66% for domain 5, and 90%, 82%, and 77% for domain 6. Generally, the NICE CPGs received significantly better clinical recommendations. However, all five evidence-based CPGs were of high methodological quality and were recommended for use in practice. Saudi Arabia should formulate its own national CPGs for diagnosis and management of perioperative hypothermia and to be published on NICE.

## Highlights

• Guidance for adapting CPGs to a given clinical practice is a realistic, practical alternative to de novo CPG development, which is a time-consuming and resource-intensive approach.
• Some nations, particularly those with low- and middle-income economies, have chosen to employ CPG adaptation, rather than developing new evidence-based practice programs.
• Several formal adaptation approaches are currently available, and they may be tailored further to suit particular circumstances. Evaluations like the one described in the present study should provide guidance for appropriate CPG adaptation or development efforts, particularly for organizations with little expertise with the AGREE II instrument.
• The current critical appraisal emphasized the importance of clinicians performing quality assessments of CPGs to ensure transparency and strength in the CPG development process, in accordance with international CPG standards. Moreover, our findings will support the provision of best practices for POH. Based on our findings, we propose that anesthesiologists should include an AGREE II review of CPGs in their capacity development strategies.

## 1 Introduction

Perioperative hypothermia (POH) is a life-threatening complication of anesthesia. POH occurs when the core body temperature falls below 36.0 °C. During surgical procedures and sedation, temperature homeostasis is disturbed, and the inter-threshold range changes from 0.4 °C to 4.0 °C; thus, the body cannot respond efficiently to heat loss by vasoconstriction or shivering. Older and very young patients are prone to temperature regulation disturbances, due to senile and immature physiological mechanisms, respectively. POH is relatively rare, because sensitive defense mechanisms prevent heat loss, unless internal or external factors intervene. In Saudi Arabia, a national prospective study conducted in 2003 found that, among 3886 patients that underwent general anesthesia for surgical procedures, the estimated POH incidence was 1.54%. Hypothermia causes a variety of negative effects, including a slowed metabolism, histotoxic hypoxia, myocardial insufficiency, delayed recovery, neuromuscular blockade, surgical site infection, postoperative shivering, disseminated intravascular coagulopathy, delayed wound healing, and patient dissatisfaction. POH is easily prevented and managed with precautions, like a warm theater (not <21 °C), warm transfusion, pre-operatively warming the patient for 30 min, and irrigation with warm saline (38–40 °C) intraoperatively. POH should be managed strictly and rapidly, according to pre-determined steps and guidelines. Various treatment modalities for managing POH have been established by societies, academies, and organizations. However, inconsistencies have led to confusion and discrepancies among schools and care givers. This situation has motivated the scientific community to establish guidelines for reducing variations in treatment, and thus, eliminating the possibility of errors. Currently, there is no national Clinical Practice Guideline (CPG) in Saudi Arabia for the care of patients with POH.

The American National Academies of Health and Medicine Division has defined CPGs as “reports that comprise recommendations envisioned to improve patient care and are well-versed by a systematic review of evidence and an evaluation of the harms and benefits of various care options” [1]. The Appraisal of Guidelines for Research and Evaluation (AGREE II) is an approved scientific quality assessment tool for appraising and comparing different CPGs. This tool evaluates CPGs according to certain criteria and determines which CPG is most reliable and complete [2]. The AGREE II is the gold standard for the critical appraisal of quality assessments or CPGs. The original AGREE tool was published in 2003, but it was recently revised in 2017 by the AGREE enterprise [3]. Thus, the AGREE II is a verified, quantitative instrument that has been mentioned in over 1013 papers and is supported by a number of healthcare organizations [4]. AGREE II outlines the stakeholders that CPGs must address to enhance quality. As a result, AGREE II assures the predicted trustworthiness of CPGs and their beneficial influence on healthcare outcomes [5].

This systematic review aimed to evaluate and critically appraise the most popular and recently published CPGs for POH management and compare them via AGREE II. Then, we analyzed the agreement among the four independent anesthesiologists that rated the CPGs, as a part of the CPG revision program.

## 2 Methods

We published the proposal for this research in the international Prospective Registry of Systematic Reviews (PROSPERO: https://www.crd.york.ac.uk/PROSPERO/display-record.php?RecordID=44439786) [6]. This review was performed according to the PRISMA-2020 statement. The format is consistent with the PRISMA criteria to provide transparency in why the review was conducted, what we did, and what we found [7]. We submitted our research to the research registry: www.researchregistry.com, with the unique identifying number: “reviewregistry1357” [8].

### 2.1 Inclusion and exclusion criteria

Four investigators independently reviewed the literature and retrieved all relevant studies and CPGs; according to the preset inclusion criteria, studies had to be:

1 “evidence-based”, clear, detailed documentaries that explained advanced methodology;
2 published in the English language;
3 attained from novel sources and de-novo databases;
4 national/international in scope; and
5 updated, edited, published, printed, or written between Jan 1, 2010 and Dec 31, 2021.

We repeated the searching process to identify more relevant CPGs, and we included:

6 CPGs published over the past ten years (2011–2021), according to the updates mentioned in the CPG handbooks; and
7 studies published by an institution or society or had “group authorship” in a CPG database or peer-reviewed journal [9,18]

Studies were excluded from the review when they were:

1 CPGs published before 2011;
2 written in a non-English language;

3- adapted from other source CPG(s);

4 presented as consensus or expert-based statements; or
5 written by a single author [18,19].

### 2.2 Search strategy and study selection

We searched several bibliographic electronic databases, including Medline-PubMed, Google Scholar, EBSCO, DynaMed Plus “USA”. We also searched international CPG databases, including: ECRI, Institute-Guidelines-Trust, the National-Institute-of-Health and Care-Excellence (NICE/UK), Guidelines-International-Network, the International-guideline-library, the Scottish-Intercollegiate-Guidelines-Network, and the National-Health and Medical-Research-Council of Australia. Furthermore, we searched electronic databases of national and international non-governmental organizations, non-profit organizations, and societies concerned with anesthesia disorders, like POH, including; the American Society of Anesthesiologists, The Royal College of Anesthetists, The Australian and New Zealand College of Anesthetists, The International Society for Anesthetic Pharmacology, Saudi Anesthesia Society, Saudi Society for Obesity and Bariatric Anesthesia, and Pan Arab Federation of Societies of Anesthesia, Intensive Care, and Pain Management.

We used the following keywords: “hypothermia” AND “body temperature” OR “warming’’ (‘‘prewarming’’, ‘‘warming techniques’’, ‘‘warming devices”, “warming system”, ‘‘active warming’’), ‘‘fall in temperature’’ (‘‘coldness”, “temperature drop’’, ‘‘surgical hypothermia’’, ‘‘intraoperative’’, ‘‘perioperative’’, ‘‘postoperative’’, ‘‘low temperature’’), AND “guidelines,” “practice-guidelines”, “clinical-practice-guidelines”, “practice-parameter”, “guidance”, OR “recommendations”.

The PubMed electronic search strategy included the following terms: “hypothermia”, “body temperature” [MeSH Terms] OR (“warming” [Title] AND “prewarming” [Title] AND “warming techniques” [Title]) OR “warming devices” [Title] OR (“warming system” [Title] AND “active warming” [Title] AND “fall in temperature” [Title]) OR “coldness” [Title]) AND “temperature drop” [Title] AND (“surgical hypothermia” [MeSH Terms] OR “intraoperative” [Title]) OR (“perioperative” [MeSH Terms] OR (“postoperative” [Title] AND “low temperature” [Title]) OR “rewarming” [Title]) AND (“guidelines” [Publication-Type] OR “guidelines as a topic” [MeSH Terms] OR “guideline” [Title]) AND (Practice Guidelines [publication type] AND (“2011/01/01” [Pub DATE]: “2021/12/31” [PDAT]) AND “humans” [MeSH-Terms]) AND (“practice-guidelines” [Filter]).

To ensure the provision of the process of CPG eligibility criteria, we applied a model that included the Patient Population, Interventions, Professionals, Outcomes, and Healthcare Setting (PPIPOHS). Four investigators (JH, KW, JF, and HG) independently screened the abstracts and titles of all studies and CPGs to identify those that fulfilled the inclusion criteria. Three other investigators reviewed the screening process (GH, SK, and JJ). After retrieving and appraising the full-text CPGs or “links” to any accessible online papers or websites, the authors held a focus-group discussion to resolve any conflicts of interest, arguments, or discrepancies [10].

### 2.3 Critical appraisal of CPGs with the AGREE II instrument

The AGREE II tool/instrument (www.agreetrust.org) comprised 23 items categorized in six quality domains:

1 Scope and purpose
2 Stakeholder involvement
3 Rigor of development
4 Clarity of presentation
5 Applicability
6 Editorial independence

Each investigator independently evaluated and assessed CPGs for each item. Items were scored on a Likert scale from one to seven, where one was “very poor performance” and seven was “excellent performance”. We used the online platform, My AGREE PLUS II, where an appraisal group can assemble the individual scores for each item and calculate a cumulative score, including comments, and domain ratings. Each investigator (rater) had relevant qualifications and experience in the field of anesthesia with a subspecialty in the management and diagnosis of perioperative hypothermia. The experience of the raters ranged from 17 to 35 years. The raters participated in training for the AGREE critical appraisal process by attending capacity-building sessions supervised by a clinical research methodologist. The methodologist trained the raters with hands-on courses on how to answer the AGREE and how to understand its concepts and standards. Each investigator scored an assigned test CPG. Then, they critically appraised the five CPGs included in this study [11].

All investigators fully reviewed the updates, CPG reports, relevant supp. Links, and information relevant to CPG methods and tools. Each AGREE II item had a “Comments” section, where the AGREE evaluators were asked to provide their reasoning for their ratings [12]. When wide disparities occurred in assessors' scores for an item or question (i.e., a difference of more than three points), the individuals with outlier scores were asked to re-assess the questions, after a conversation with the full group [13]. The My AGREE PLUS platform automatically determined the standardized AGREE domain scores or ratings (percentages) [14].

For each AGREE standardized domain score or rating, a cut-off value of 70% was established. After the assessment, we concentrated on the scores for domains 3 and 5 to facilitate the filtering and final evaluation of the reporting quality of the included CPGs [15]. Previous studies have implemented similar cut-off values. In addition to the six AGREE II domains, the evidence and references cited to support the included CPGs were checked for systematic reviews or meta-analyses, particularly Cochrane reviews [16].

This evaluation was reported according to the PRISMA statement (https://www.sciencedirect.com/science/article/pii/S1743919121000406?via%3Dihub). Accordingly, we included an appropriate flow diagram and checklist. This research did not require any patient or public participation [17].

### 2.4 Inter-rater analysis

Agreement between raters was determined with inter-rater reliability assessment tests (IRR). The percent IRR was used to determine the degree of agreement among the four raters. We also used the IRR to determine the percent of agreement among the first overall assessments performed with the AGREE II instrument, for each question (or item) in each standardized domain of the four CPGs included in this study. Furthermore, we calculated the intra-class correlation coefficient (ICC) to evaluate the consistency in the ratings or capacity of the datasets obtained as clusters or grouped into clusters, including the second overall assessment (i.e., recommendation for using the CPG).

In studies with more than two raters, one of the most commonly used IRR systems is the ICC. A high ICC (or Kappa) score (i.e., around 1) suggests that standards from the same set are very similar. A low Kappa value (around 0) suggests that standards from the same set are not identical. The one-way random analysis of variance was used. The data analyses were performed with SPSS Statistics, version 21 [18].

Due to the variety of numerical data gathered from the groups or clusters, we used the ICC to establish the repeatability of the data and to determine how closely the peers matched, in terms of certain qualities or attributes. The data were received from ordered scales; consequently, agreement between two ordinal scale classifications was tested with a weighted Kappa (Quadratic Weights) [19].

Because the difference between the first and second categories had the same importance as the difference between the second and third categories, and so on, we utilized linear weights. We quantified agreement with the K statistic, where K = 1 indicated complete agreement between categorization systems; K = 0 indicated agreement no greater than chance; and K < 0 indicated agreement less than chance. Based on the K value, the degree of agreement was rated as follows: 0.20 (poor), 0.21–0.30 (fair), 0.31–0.40 (moderate), 0.41–0.60 (good), 0.61–0.80 (very good), and 0.81–1.00. (excellent) [20].

## 3 Results

### 3.1 Identification of psoriasis management CPGs

**Fig. 1:**
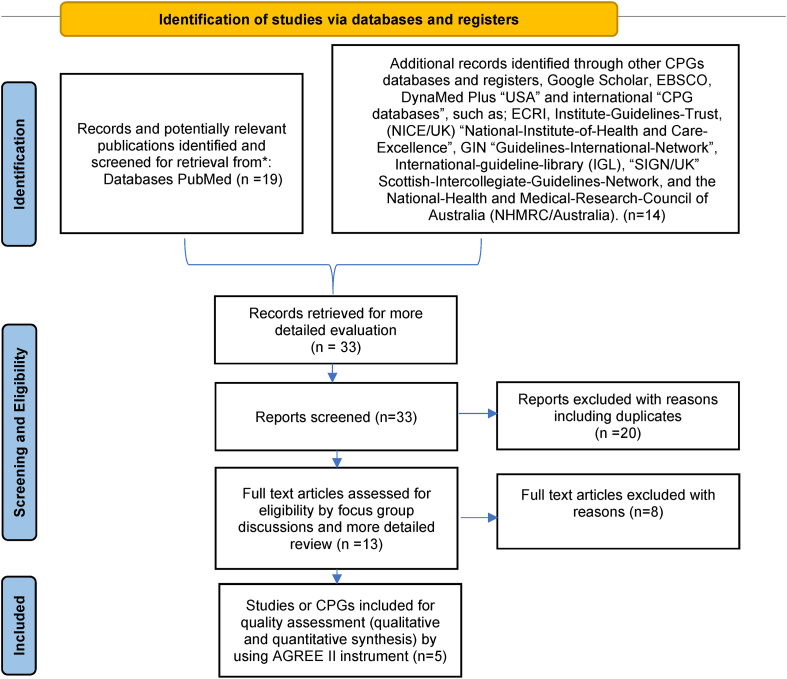
PRISMA flow diagram. Systematically searching and selecting the clinical practice guidelines for the management of psoriasis. From: Moher D, Liberati A, Tetzlaff J, Altman DG, The PRISMA Group (2009). Preferred Reporting Items for Systematic Reviews and Meta-Analyses: The PRISMA Statement. PLoS Med 6(7): e1000097. doi:https://doi.org/10.1371/journal.pmed1000097. For more information, visit www.prisma-statement.org.

Initially, the screening and reviewing process identified 35 reports. However, we excluded 31 reports that did not meet the inclusion criteria. The selection process is summarized in the PRISMA diagram (Fig. 1) [21,22]. Finally, five CPGs were sufficiently recent and conformed to the pre-determined inclusion criteria and PIPPOH model (Fig. 1). The five CPGs were: (*i*) National Institute for Health and Care Excellence (NICE-2016) for Peri-operative hypothermia: Assessment and Management [23]; (*ii*) American Society of Peri-Anesthesia Nurses/Agency for Health Care Research and Quality: clinical guideline for the prevention of unplanned perioperative hypothermia (ASPAN/AHRQ-2006) [24]; (*iii*) The University of Southern Mississippi: Temperature Guideline to Decrease Intraoperative Hypothermia in Patients Undergoing General Anesthesia (USM/CPG-2017) [25]; (*iv*) University Assistance Complex of Salamanca Clinical practice guideline: “Unintentional perioperative hypothermia” (UACS/CPG-2018) [26]; and (*v*) Justus-Liebig university of Giessen/Clinical practice guideline: “Preventing inadvertent perioperative hypothermia” (UKGM/CPG-2015) [27].

Table 1 shows the characteristics of the five eligible CPGs. The CPGs were established by reference-professional-specialized organizations based in developed countries: one from the UK (NICE-2016), two from the USA (ASPAN/AHRQ-2006 and USM/CPG-2017), one from Spain (UACS/CPG-2018), and one from Germany (UKGM/CPG-2015). All five organizations were considered reputable international “evidence-based” healthcare development institutions [[33], [34], [35], [36]].

**Table 1:** Characteristics of the included CPGs.

Title	Year of publication	Country	Level of development	Organization (short name)	Total number of references
National Institute for Health and Care Excellence (NICE-2016) for Peri-operative hypothermia: Assessment and Management [21]	2016 (Reaffirmed 2017)	United Kingdom	National	National Institute of Health and Care Excellence (NICE)	36 (one NCSR)
American Society of Peri-Anesthesia Nurses/Agency for Health Care Research and Quality clinical guideline for the prevention of unplanned perioperative hypothermia (ASPAN/AHRQ-2006) [22],	2006 (minor update in 2008)	United States	National	American Society of Peri-Anesthesia Nurses/Agency for Health Care Research and Quality (ASPAN/AHRQ-2006),	87 (one NCSR) (reviewed and excluded NCSR and CSRs were not counted)
The University of Southern Mississippi/Temperature Guideline to Decrease Intraoperative Hypothermia in Patients Undergoing General Anesthesia (USM/CPG-2017) [23],	2017 (updated 2019)	United States	National	The University of Southern Mississippi (USM/CPG-2017),	70 (one NCSR, 3 CSR)
University Assistance Complex of Salamanca/Clinical practice guideline “Unintentional perioperative hypothermia” (UACS/CPG-2018) [24],	2018 (updated 2019)	Spain	National	University Assistance Complex of Salamanca (UACS/CPG-2018),	328 (4 NCSR, one CSR)
Justus-Liebig university of Giessen/Clinical practice guideline “Preventing inadvertent perioperative hypothermia” (UKGM/CPG-2015) [25].	2015 (updated 2016)	Germany	National	Justus-Liebig university of Giessen (UKGM/CPG-2015).	8 (one NCSR)

Abbreviations: *CPG* clinical practice guideline; *CSR* Cochrane systematic review; *NCSR* Non-Cochrane systematic review. National Institute for Health and Care Excellence (NICE-2016) for Peri-operative hypothermia: Assessment and Management, American Society of Peri-Anesthesia Nurses/Agency for Health Care Research and Quality clinical guideline for the prevention of unplanned perioperative hypothermia (ASPAN/AHRQ-2006), The University of Southern Mississippi/Temperature Guideline to Decrease Intraoperative Hypothermia in Patients Undergoing General Anesthesia (USM/CPG-2017), University Assistance Complex of Salamanca/Clinical practice guideline “Unintentional perioperative hypothermia” (UACS/CPG-2018), Justus-Liebig university of Giessen/Clinical practice guideline “Preventing inadvertent perioperative hypothermia” (UKGM/CPG-2015).

### 3.2 Reporting the quality of CKD management CPGs

**Fig. 2:**
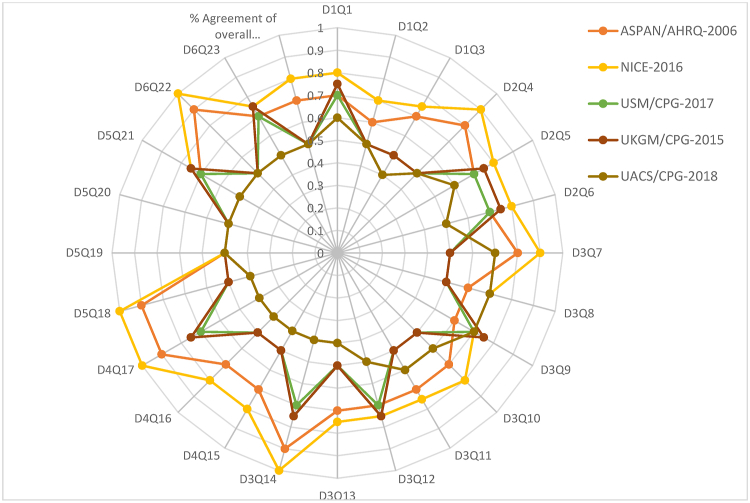
Using a Radar chart to map the AGREE II 23-questions, 6-domains, and the first overall assessment for eligible appraised clinical guidelines. Abbreviations: National Institute for Health and Care Excellence (NICE-2016) for Peri-operative hypothermia: Assessment and Management, American Society of Peri-Anesthesia Nurses/Agency for Health Care Research and Quality clinical guideline for the prevention of unplanned perioperative hypothermia (ASPAN/AHRQ-2006), The University of Southern Mississippi/Temperature Guideline to Decrease Intraoperative Hypothermia in Patients Undergoing General Anesthesia (USM/CPG-2017), University Assistance Complex of Salamanca/Clinical practice guideline “Unintentional perioperative hypothermia” (UACS/CPG-2018), Justus-Liebig university of Giessen/Clinical practice guideline “Preventing inadvertent perioperative hypothermia” (UKGM/CPG-2015).; AGREE II Appraisal of Guidelines for Research and Evaluation II; CPG clinical practice guideline or guidance. AGREE: Appraisal of Guidelines for Research and Evaluation, CPG: clinical practice guideline or guidance.

**Fig. 3:**
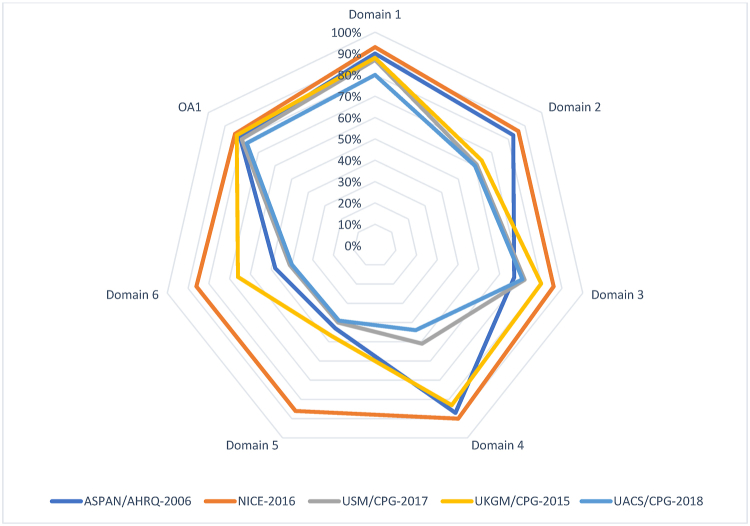
Radar map of the AGREE II final standardized domain scores for eligible appraised clinical guidelines. Abbreviations: National Institute for Health and Care Excellence (NICE-2016) for Peri-operative hypothermia: Assessment and Management, American Society of Peri-Anesthesia Nurses/Agency for Health Care Research and Quality clinical guideline for the prevention of unplanned perioperative hypothermia (ASPAN/AHRQ-2006), The University of Southern Mississippi/Temperature Guideline to Decrease Intraoperative Hypothermia in Patients Undergoing General Anesthesia (USM/CPG-2017), University Assistance Complex of Salamanca/Clinical practice guideline “Unintentional perioperative hypothermia” (UACS/CPG-2018), Justus-Liebig university of Giessen/Clinical practice guideline “Preventing inadvertent perioperative hypothermia” (UKGM/CPG-2015).; AGREE II Appraisal of Guidelines for Research and Evaluation II; CPG clinical practice guideline or guidance. AGREE: Appraisal of Guidelines for Research and Evaluation, CPG: clinical practice guideline or guidance.

The consistent domain assessments of AGREE II are shown in Table 2, and the explanations given by the evaluators are shown in Table 3. The standardized scores were above 70% for all five CPGs in domain 1 (Scope and Purpose), for only two CPGs in domain 2 (Stakeholder involvement), for two CPGs in domain 3 (Rigor of development), for three CPGs in domain 4 (Clarity of presentation), for only one CPG in domain 5 (Applicability), and only one CPG in domain 6 (Editorial independence). The scores for the AGREE II first overall assessment ranged from 77% to 84%. All CPGs ratings more than 70%, conformed with their high ratings in all domains. The AGREE II premeditated domain ratings are presented in Fig. 2, Fig. 3. The radar chart illustrates the final ratings for every CPG, shown as percentages (%), for the “six” domains (Fig. 2) and for each of the 23 questions in AGREE II (Fig. 3).

**Table 2:** AGREE II consistent domain ratings for the five CPGs.

CPGs/AGREE II Domains-standardized scores (%)	NICE-2016 [21]	ASPAN/AHRQ-2006 [22]	USM/CPG-2017 [23]	UKGM/CPG-2015 [24]	UACS/CPG-2018 [25]
Domain 1. Scope and purpose Items 1–3: Objectives; Health question(s); Population (patients, public, etc.).	93%	90%	88%	87%	80%
Domain 2. Stakeholder Involvement Items 4–6: Group Membership; Target population preferences and views; Target users	86%	83%	64%	61%	60%
Domain 3. Rigor of development Items 7–14: Search methods; Evidence selection criteria; Strengths and limitations of the evidence; Formulation of recommendations; Consideration of benefits and harms; Link between recommendations and evidence; External review; Updating procedure	94%	81%	70%	66%	65%
Domain 4. Clarity and presentation Items 15–17: Specific and unambiguous recommendations; Management options; Identifiable key recommendations	90%	87%	83%	51%	44%
Domain 5. Applicability Items 18–21: Facilitators and barriers to application; Implementation advice/tools; Resource implications; Monitoring/auditing criteria	91%	87%	66%	61%	60%
Domain 6. Editorial independence Items 22, 23: Funding body; Competing interests	90%	82%	77%	71%	70%
Overall Assessment 1 (Overall quality)	81%	84%	80%	83%	77%
Overall Assessment 2	Yes (n = 2); Yes with	Yes (n = 3); Yes with	Yes (n = 1); Yes with	Yes (n = 2); Yes with	Yes (n = 1); Yes with
(Recommend the CPG for use by the four appraisers)	modifications (n = 2); No (n = 0).	modifications (n = 1); No (n = 0).	modifications (n = 3); No (n = 0).	modifications (n = 2); No (n = 0).	modifications (n = 3); No (n = 0).

Abbreviations: National Institute for Health and Care Excellence (NICE-2016) for Peri-operative hypothermia: Assessment and Management, American Society of Peri-Anesthesia Nurses/Agency for Health Care Research and Quality clinical guideline for the prevention of unplanned perioperative hypothermia (ASPAN/AHRQ-2006), The University of Southern Mississippi/Temperature Guideline to Decrease Intraoperative Hypothermia in Patients Undergoing General Anesthesia (USM/CPG-2017), University Assistance Complex of Salamanca/Clinical practice guideline “Unintentional perioperative hypothermia” (UACS/CPG-2018), Justus-Liebig university of Giessen/Clinical practice guideline “Preventing inadvertent perioperative hypothermia” (UKGM/CPG-2015).*AGREE II* Appraisal of Guidelines for Research and Evaluation II; *CPG* clinical practice guideline or guidance.

**Table 3:** Critics’ commentaries on the five “CPGs” prearranged according to the consistent domains in “AGREE II”.

AGREE II Domain	Strengths	Limitations
Domain 1. Scope and purpose	•Objectives, purpose, health intent, clinical questions, and patient population were clearly mentioned in the CPG full document or the website using the PICO model. ^β^	•Target users were general rather than specific ^α^
Domain 2. Stakeholder Involvement	•GDG members' names, specialties, institutions, and geographical locations were clearly mentioned and easy to find. GDG included methodologist(s). •GDG included members from relevant professional groups including patient representatives. **	•GDG disciplines and roles were not clearly mentioned. ^α^ •GDG was missing some key disciplines (e.g. pharmacists and nurses).^#^ •Lack of adequate and clear descriptions of patient participation or preferences and target users.^#^
Domain 3. Rigor of development	•Detailed evidence search keywords were mentioned ** •The GRADE (Grading of Recommendations Assessment, Development and Evaluation) approach to assess the quality of evidence was utilized** •Recommendations include health benefits, harms, and side effects of recommendations with or without a discussion of their trade-offs * •All recommendations were linked to their relevant primary source of evidence* •Lists and processes of external review were clearly reported and easy to find * •Updating was clearly mentioned * * •This domain was well-addressed in most included CPGs, where key recommendations were specific, unambiguous, and easily identifiable in all CPGs ^β,^ *^,^ **	•Lack of detailed search strategy.^#^ •Strengths and limitations of the body of evidence (evidence tables) were not clearly reported.^#^ •Lack of detailed process for formulation of the recommendations, and discussion of a trade-off be- tween harms and benefits. ^α^ •Details and methods of the external review process and outcomes were not clearly reported.^#^
Domain 4. Clarity and presentation	•Some facilitators and barriers to implementations and clinical governance issues were discussed ^β,^ *^,^ ** •A package of CPG Implementation tools was provided like educational tools, protocols, summary document, patient, information clinical algorithm or pathway, baseline assessment sheet, Mobile App. **	•Review and update process was not reported. ^α^ •Management of Crisis were not highlighted. ^α^
Domain 5. Applicability	•Quality standards, measures, indicators, and/or clinical audit criteria were provided. ^β^ •A formal economic analysis was conducted. *, **	•Facilitators and barriers to implementations were not explicitly mentioned.^#^ •Implementation tools were not provided.^#^ •Quality measures or key performance indicators were not provided.^#^ •No formal economic analysis was conducted.^#^ •Funding and influence statements were not clearly reported.^#^ •No DCOI statements were provided. ^α^
Domain 6. Editorial independence	•Funding with or without an influence statement was mentioned. •DCOI statements were clearly provided.	

Abbreviations: National Institute for Health and Care Excellence (NICE-2016) for Peri-operative hypothermia: Assessment and Management, American Society of Peri-Anesthesia Nurses/Agency for Health Care Research and Quality clinical guideline for the prevention of unplanned perioperative hypothermia (ASPAN/AHRQ-2006), The University of Southern Mississippi/Temperature Guideline to Decrease Intraoperative Hypothermia in Patients Undergoing General Anesthesia (USM/CPG-2017), University Assistance Complex of Salamanca/Clinical practice guideline “Unintentional perioperative hypothermia” (UACS/CPG-2018), Justus-Liebig university of Giessen/Clinical practice guideline “Preventing inadvertent perioperative hypothermia” (UKGM/CPG-2015).^α^ AGREE II Appraisal of Guidelines for Research and Evaluation II; CPG clinical practice guideline or guidance.

### 3.3 CPGs recommended for use in clinical practice

The second overall assessment showed that the raters agreed on their recommendations for applying the CPGs in practice. However, all the recommendations included modifications and adjustments. Table 3 summarizes the strengths and limitations of the included CPGs, based on a consensus of the comments made by the CPG appraisers for each item in the AGREE II assessment. In their references, all five CPGs were mentioned as systematic reviews. The NICE-2016 CPG mentioned the most systematic reviews (n = 6). Among those reviews, five (83%) were Cochrane reviews. Overall, the various treatment choices for POH were comparable in all five CPGs (Table 4).

**Table 4:** Summary of key recommendations in the five CPGs from ASPAN/AHRQ-2006[22], NICE-2016[21], USM/CPG-2017[23], UKGM/CPG-2015[24], UACS/CPG-2018 [25].

CPGs/Recommendations	ASPAN/AHRQ-2006 [22]	NICE-2016 [21]	USM/CPG-2017 [23]	UKGM/CPG-2015 [24]	UACS/CPG-2018 [25]
Target users	Not mentioned	Mentioned	Not Mentioned	Not mentioned	Not mentioned
Disciplines and roles	Not mentioned	Mentioned	Not mentioned	Mentioned	Not mentioned
Key disciplines	Not Mentioned	Mentioned	Not Mentioned	Not mentioned	Not Mentioned
Clear descriptions of patient participation	Mentioned	Mentioned	Not Mentioned	Mentioned	Mentioned
Search strategy	Not mentioned	Mentioned	Not Mentioned	Mentioned	Not mentioned
Evidence tables	Mentioned	Mentioned	Not mentioned	Not Mentioned.	Mentioned
Formulation of the recommendations	Mentioned	Mentioned	Mentioned	Mentioned	Mentioned
External review process	Mentioned	Mentioned	Not mentioned	Mentioned	Mentioned
Review and update process	Mentioned	Not mentioned	Mentioned	Mentioned	Mentioned
Management of Crisis	Mentioned	Not mentioned	Mentioned	Mentioned	Not mentioned
Barriers to implementations	Not mentioned	Not mentioned	Not Mentioned.	Mentioned	Mentioned
Implementation tools	Mentioned	Not mentioned	Mentioned	Mentioned	Not mentioned
Quality measures	Not mentioned	Not mentioned	Mentioned	Not mentioned	Not mentioned
Economic analysis	Mentioned	Mentioned	Mentioned	Mentioned	Not mentioned
Funding and influence statements	Mentioned	Not mentioned	Mentioned	Mentioned	Not mentioned
DCOI statements	Mentioned	Not mentioned	Mentioned	Not Mentioned.	Not Mentioned
Clinical questions	Not mentioned	Not mentioned	Not mentioned	Mentioned	Not mentioned
Health intent	Mentioned	Not mentioned	Not mentioned	Mentioned	Mentioned
Purpose	Mentioned	Mentioned	Not mentioned	Mentioned	Not mentioned
Objectives	Mentioned	Mentioned	Mentioned	Mentioned	Mentioned

Abbreviations: National Institute for Health and Care Excellence (NICE-2016) for Peri-operative hypothermia: Assessment and Management, American Society of Peri-Anesthesia Nurses/Agency for Health Care Research and Quality clinical guideline for the prevention of unplanned perioperative hypothermia (ASPAN/AHRQ-2006), The University of Southern Mississippi/Temperature Guideline to Decrease Intraoperative Hypothermia in Patients Undergoing General Anesthesia (USM/CPG-2017), University Assistance Complex of Salamanca/Clinical practice guideline “Unintentional perioperative hypothermia” (UACS/CPG-2018), Justus-Liebig university of Giessen/Clinical practice guideline “Preventing inadvertent perioperative hypothermia” (UKGM/CPG-2015). AGREE II Appraisal of Guidelines for Research and Evaluation II; CPG clinical practice guideline or guidance. AGREE: Appraisal of Guidelines for Research and Evaluation, CPG: clinical practice guideline or guidance.

### 3.4 Percent agreement and inter-rater analysis

The IRR tests revealed a high level of agreement among the four raters for every question in every domain for the five CPGs. The percent agreement among raters for the first overall assessment is shown in Fig. 2. The majority of K values were between 0.50 and 1.00, which indicated good to outstanding agreement among raters. Only two assessments in the UACS/CPG exhibited inadequate agreement strength [K = 0.00]: question 6 in domain 2 [D2Q6] and question 8 in domain 3 [D3Q8]. Table 5 shows the frequencies of reaching different degrees of agreement among the raters for all five CPGs. For example, of the 24 questions in the UKGM CPG, excellent agreement [K = 1.0] was reached in one, good agreement [K = 0.50] was reached in 16, very good agreement [K = 0.60–0.80] was reached in five, and poor agreement [K = 0.00] was reached in two. Moreover, good to excellent agreement between raters [K = 0.50] was reached for the first overall assessments for all CPGs.

**Table 5:** Cataloguing of the forte of agreement among the four investigators for the five CPGs.

	Poor	Fair	Good	Very good	Excellent	Overall assessment 1
(NICE-2016) [21]	0	0	12	9	3	Excellent
(USM/CPG-2017) [22],	0	0	16	7	1	Good
(UKGM/CPG-2015) [23].	2	0	16	5	1	Good
(ASPAN/AHRQ-2006) [24],	0	0	13	9	2	Very Good
(UACS/CPG-2018) [25],	3	0	15	5	1	Good

Abbreviations: National Institute for Health and Care Excellence (NICE-2016) for Peri-operative hypothermia: Assessment and Management, American Society of Peri-Anesthesia Nurses/Agency for Health Care Research and Quality clinical guideline for the prevention of unplanned perioperative hypothermia (ASPAN/AHRQ-2006), The University of Southern Mississippi/Temperature Guideline to Decrease Intraoperative Hypothermia in Patients Undergoing General Anesthesia (USM/CPG-2017), University Assistance Complex of Salamanca/Clinical practice guideline “Unintentional perioperative hypothermia” (UACS/CPG-2018), Justus-Liebig university of Giessen/Clinical practice guideline “Preventing inadvertent perioperative hypothermia” (UKGM/CPG-2015).; AGREE II Appraisal of Guidelines for Research and Evaluation II; CPG clinical practice guideline or guidance. AGREE: Appraisal of Guidelines for Research and Evaluation, CPG: clinical practice guideline or guidance.

The second overall assessment provided recommendations for the five CPGs. We found poor agreement among the raters, with K = 0.1670, standard error = 0.1380 (95% confidence interval: 0.1030–0.4370); and the weighted was K = 0.0770.

## 4 Discussion

This study was the first to use the “AGREE II” instrument/tool to perform a comprehensive assessment of the quality of newly published CPGs for managing POH. The AGREE II results revealed several opportunities for improving the methodological rigor of the CPGs. One CPG (UACS) showed major gaps in development rigor (domain 3), which is the largest and most important domain. Three CPGs showed room for improvement in their applicability (domain 5). This study highlighted the importance of these two areas. The NICE-2016 CPG received the highest levels of reviewer agreement. The clinical advice provided in the five CPGs showed some similarities and some variability (Table 4).

The NICE-2016, ASPAN/AHRQ-2006, and USM/CPG-2017 mentioned details about blankets. In contrast, the UKGM/CPG-2015 and UACS/CPG-2018 did not mention blankets. Several types of blankets were mentioned: (*i*) blankets made of cotton or a cotton cover can be used with or without pre-warming. (*ii*) Disposable blankets made of Mediwrap®, which are constructed of three layers of fabric: the outside layer is waterproof, the intermediate layer is reflective, and the layer in contact with the patient is smooth, soft, and absorbent. The blanket can be warmed actively with a forced air system, which comprises a machine that creates warm air and passes it through a pipe connected to the disposable device. The disposable device may be utilized beneath the patient's body or it can cover various parts of the body; for example, the whole body, the upper body, or the lower body. (*iii*) A water-circulating mattress, which contains hot water that flows from a water-heating unit. This system is placed underneath the patient and can be cleaned and reused. (*iv*) A mattress made of carbon fiber that resists warmth, which can be placed under the patient and linked to an electrical warming unit. After cleaning, it is re-used. For example, heating blankets are non-disposable covers made of carbon fiber. They can cover the entire body or particular sections of the patient (trunk, arms, or legs). The cover is linked to a heating unit that runs on electricity.

In three CPGs (USM/CPG-2017, UACS/CPG-2018, and UKGM/CPG-2015), disagreement was noted concerning the lack of clearly specified recommendations for general screening, ongoing monitoring, temporary discontinuation, and reinitiating therapy. Compared to the two other CPGs, which featured broad advice for POH, one CPG (NICE-2016) included more details about ongoing monitoring. The NICE-2016 consistently showed higher ratings than the other CPGs in all domains. After examining the other four CPGs and considering the proper rigor, consistently high ratings, and clinical relevance, we opted to use the NICE-2016 CPG and all the recommendations for improvement in our clinical practice. This comprehensive, impartial evaluation of the various CPGs may facilitate the choice of accepting or altering the CPGs for clinical practice.

Our results demonstrated that the AGREE II CPG evaluation was correct. The four assessors that used AGREE II to evaluate the five CPGs showed excellent/very good inter-rater agreement. The proposed method could be used as a model for similar systematic reviews and evaluations of CPG quality. Furthermore, the statistical analysis performed in this study demonstrated the usefulness of the AGREE II instrument as a tool for the critical evaluation of CPGs; it provided benefit to the assessors without sacrificing assessment quality. Inexperienced personnel or non-professional reviewers could not have achieved a comparable consensus about the clinical features and characteristics of the CPGs. which might have influenced the decision of the provision of treatment of POH.

To the best of our knowledge, no previous systematic review has evaluated CPG quality, except one systematic review, which evaluated a CPG on perioperative care; however, that review used the GRADE instrument, rather than the AGREE II. They did not draw any conclusions, because only a protocol was published. Moreover, they did not include any of the five CPGs that we evaluated [28]. General screening, ongoing monitoring, temporary discontinuation, and reinitiating therapy were among the high priority health topics [29]. Some previous studies have discovered several gaps among CPGs, such as differences or discrepancies, a lack of evidence, and inconsistencies in clinical recommendations; additionally, a few commonalities and similarities were observed among different studies concerning recommendations for improving CPG variability [[33], [34], [35], [36]].

### 4.1 Strengths and limitations

One strength of the present study was that the evaluation was carried out by a clinical team specialized in anesthesia and led by an experienced CPG methodologist. This team provided another layer of strength to the AGREE II assessment. Other strengths of this study were: (*i*) the use of an international, rigorously structured, validated CPG appraisal tool: the AGREE II instrument; (*ii*) the use of four raters for appraising each CPG; (*iii*) the thoroughness of our search across several databases; and (*iv*) the statistical analysis of inter-rater differences.

Care providers must be encouraged to embrace and integrate evidence-based and eminence-based healthcare concepts into their everyday practice with ongoing training and learning about high-quality CPG standards and assessment techniques [30]. The findings of this review may be utilized to design or adjust CPGs for POH. Moreover, our findings emphasized the importance of including the AGREE II criteria during capacity development, because it will assist physicians in finding and implementing CPGs for use in everyday practice [[31], [32], [33], [34], [35]].

This study had some limitations. First, the AGREE II instrument had some drawbacks. Some of these drawbacks have been addressed in the newly created “AGREE-REX” (Recommendation Excellence). This upgrade can assess the clinical validity of CPG recommendations. AGREE- REX has been confirmed and made publicly available on the website. Second, we used 70% as the cut-off value for standard domain ratings, despite the fact that the original AGREE II does not require a cut-off value. Nevertheless, some previous studies have adopted this cut-off value. Third, the exclusion of non-English language CPGs might have caused us to miss some relevant CPGs that were created for use in non-English speaking healthcare settings. Finally, this study focused on CPGs for the care of patients with POH, due to the known health burden posed by POH. Thus, the included CPGs predominantly focused on the management of POH (i.e. “US”-based, Germany, Spain and “UK”- based).

### 4.2 Implications for practice

Guidance for adapting CPGs to a given clinical practice is a realistic, practical alternative to de novo CPG development, which is a time-consuming and resource-intensive approach [36]. Some nations, particularly those with low- and middle-income economies, have chosen to employ CPG adaptation, rather than developing new evidence-based practice programs [37]. Several formal adaptation approaches are currently available, and they may be tailored further to suit particular circumstances. Evaluations like the one described in the present study should provide guidance for appropriate CPG adaptation or development efforts, particularly for organizations with little expertise with the AGREE II instrument.

The current critical appraisal emphasized the importance of clinicians performing quality assessments of CPGs to ensure transparency and strength in the CPG development process, in accordance with international CPG standards. Moreover, our findings will support the provision of best practices for POH. Based on our findings, we propose that anesthesiologists should include an AGREE II review of CPGs in their capacity development strategies.

## 5 Conclusion

Three evidence-based CPGs have better methodological features than the expert consensus. Our findings suggested that the CPGs included in this study could be arranged in the following order, according to quality: NICE-2016; ASPAN/AHRQ-2006; USM/CPG-2017; UKGM/CPG-2015; and UACS/CPG-2018. Based on our results, we recommend that the AGREE II criteria and the procedures used in the NICE-2016 CPG should be used as models.

## Ethical approval

IRB-KKUH-KSUMC- 982709092.

## Funding

No funding sources.

## Authors’ contributions

Babakir performed; study concept, design, data collection, data analysis, interpretation and wrote the paper.

## Registration of research studies

Name of the registry: N/A.

Unique Identifying number or registration ID: N/A.

Hyperlink to your specific registration (must be publicly accessible and will be checked): **N/A**.

## Guarantor

Leeds University.

## Availability of data and materials

All data analyzed during this study are included in the published article.

## Consent

Written and signed consent obtained.

## Provenance and peer review

Not commissioned, externally peer-reviewed.

## Declaration of competing interest

No conflicts of interest.
